# A base substitution in *OsphyC* disturbs its Interaction with OsphyB and affects flowering time and chlorophyll synthesis in rice

**DOI:** 10.1186/s12870-022-04011-y

**Published:** 2022-12-27

**Authors:** Xiaoli Lin, Yongping Huang, Yuchun Rao, Linjuan Ouyang, Dahu Zhou, Changlan Zhu, Junru Fu, Chunlian Chen, Jianhua Yin, Jianmin Bian, Haohua He, Guoxing Zou, Jie Xu

**Affiliations:** 1 Key Laboratory of Crop Physiology, Ecology and Genetic Breeding, Ministry of Education, College of Agronomy, Jiangxi Agricultural University, 330045 Nanchang, China; 2 National Engineering Laboratory of Rice (Nanchang), Rice Research Institute, Jiangxi Academy of Agricultural Sciences, 330200 Nanchang, China; 3 College of Chemistry and Life Sciences, Zhejiang Normal University, 321004 Jinhua, China

**Keywords:** Phytochrome C, Flowering time, Chlorophyll synthesis, Dimerization, Rice

## Abstract

**Background:**

Phytochromes are important photoreceptors in plants, and play essential roles in photomorphogenesis. The functions of PhyA and PhyB in plants have been fully analyzed, while those of PhyC in plant are not well understood.

**Results:**

A rice mutant, *late heading date 3* (*lhd3*), was characterized, and the gene *LHD3* was identified with a map-based cloning strategy. *LHD3* encodes phytochrome C in rice. Animo acid substitution in OsphyC disrupted its interaction with OsphyB or itself, restraining functional forms of homodimer or heterodimer formation. Compared with wild-type plants, the *lhd3* mutant exhibited delayed flowering under both LD (long-day) and SD (short-day) conditions, and delayed flowering time was positively associated with the day length via the Ehd1 pathway. In addition, *lhd3* showed a pale-green-leaf phenotype and a slower chlorophyll synthesis rate during the greening process. The transcription patterns of many key genes involved in photoperiod-mediated flowering and chlorophyll synthesis were altered in *lhd3*.

**Conclusion:**

The dimerization of OsPhyC is important for its functions in the regulation of chlorophyll synthesis and heading. Our findings will facilitate efforts to further elucidate the function and mechanism of OsphyC and during light signal transduction in rice.

**Supplementary Information:**

The online version contains supplementary material available at 10.1186/s12870-022-04011-y.

## Background

Light is not only used as source of energy for plants, also as one of the most important environmental factors that regulate multiple developmental process, such as seed germination, hypocotyl elongation, chlorophyll (Chl) synthesis, leaf shape, movement and senescence, and flowering, in higher plants [1]. In plants, several photoreceptors, such as phytochromes, flavoproteins and Ultraviolet Resistance Locus 8, are essential for sensing various light signals [2–4]. As the photoreceptors for the red and far-red regions of the visible light spectrum, plant phytochromes exist in two forms, red-light absorbing form (Pr) and far-red absorbing form (Pfr), and the former is biologically inactive [5]. Red-light absorption converts Pr form to activated Pfr, and triggers the nuclear translocation of phytochromobilin and transcriptional signaling networks, finally regulates expression of a large number of genes for diverse pathways (reviewed by Nagatani [5] and Rockwell et al. [6]).

Phytochromes are encoded by small multigene families. The five phytochrome genes in *Arabidopsis thaliana*, *AtPhyA* to *AtPhyE*, are clustered into two groups. Type I (AtPhyA) is light-labile, and its Pfr form is unstable [7]; while the Type II phytochromes (including AtPhyB, AtPhyC, AtPhyD, and AtPhyE) are light-stable and mainly function during daytime [8]. Phytochromes always function as either homo- or heterodimers. In *Arabidopsis*, AtPhyA is functional in its homodimer form, AtPhyB can form either a homodimer or heterodimer with other type II phytochromes, while AtPhyC and AtPhyE are present mostly as heterodimers [9]. In rice, there are only three phytochrome isoforms, OsphyA, OsphyB and OsphyC. Over the past decade, all single, double, and triple mutants of rice phytochromes have been isolated or constructed [10–13]. The *osphyA* mutants showed no changes to their vegetative phenotype or flowering time, but their de-etiolation and coleoptile elongation were impaired under FR light [10]. OsphyB functions in responses to red light but, unlike in *Arabidopsis*, it is not the sole red-light photoreceptor in rice [11, 14]. OsphyC seems to have minor roles in photomorphogenesis, and the *OsphyC* mutant showed no clear phenotypic differences in the seedling and vegetative growth or coleoptile and mesocotyl elongation under continuous R or FR [11, 12]. However, *osphyA/osphyC* double-mutants exhibit more severe inhibition of de-etiolation than *osphyA* single-mutant plants under continuous FR, suggesting that OsphyC coordinates the photo-sensing of FR with the assistance of other phytochromes [12]. In both *Arabidopsis* and rice, the functions of PhyC have been reported to be dependent on PhyB. OsphyC was detected at a lower concentration in *osphyB* mutants than wildtype (WT) seedlings; *osphyB/osphyC* double-mutants were no less sensitive to FR than *osphyB* mutants. These observations suggest that OsphyB somehow affects OsphyC during the photo-sensing of FR or R in rice [11, 12].

As photoreceptors, phytochromes are also important participants in the photoperiodic regulation of flowering in the rice plant. The effect of the phytochromes on rice flowering is complicated, and each phytochrome makes distinct contributions to the control of flowering time: *osphyB* mutants cause moderate early flowering under LD condition; *osphyA* mutations had few effects on flowering time [11, 13]; while the effects of OsphyC are still unclear and under debate. Firstly, the sole *osphyC* rice mutant reported has shown a different flowering phenotype in different experiments. Takano et al. found that *osphyC* mutants flowered about two weeks earlier than the WT under LD, but no difference occurred in SD conditions [11]. In another experiment, the flowering time of *osphyC* was not significantly altered under either LD or SD conditions [13]. Moreover, *phyC* mutants in different species may display contrasting flowering phenotypes [15]. Contrary to *Arabidopsis*, in wheat loss-of-function mutants of *phyC*, flowering was delayed under both LD and SD conditions, being much more significant under LD [16, 17]. Delayed flowering phenotype of *phyC* was also found in some other species, such as *Brachypodium distachyon* [18]. Therefore, PhyC plays complex roles in photoperiodic flowering pathway, and the molecular mechanism behind its role is always ignored by researchers because PhyC is reported function in PhyB-dependent manner.

In this study, we isolated and characterized a *late-heading-date3* (*lhd3*) rice mutant, that exhibited pale green leaves and delayed flowering time. Map-based cloning revealed that *lhd3* harbored a single-base substitution in the coding region of *OsphyC*. On the expression analysis of *OsphyC* and related genes, we confirmed that OsphyC plays important roles in rice photomorphogenic processes, such as Chl synthesis and the photoperiodic control of flowering.

## Results

### The phenotype of *lhd3*

**Fig. 1:**
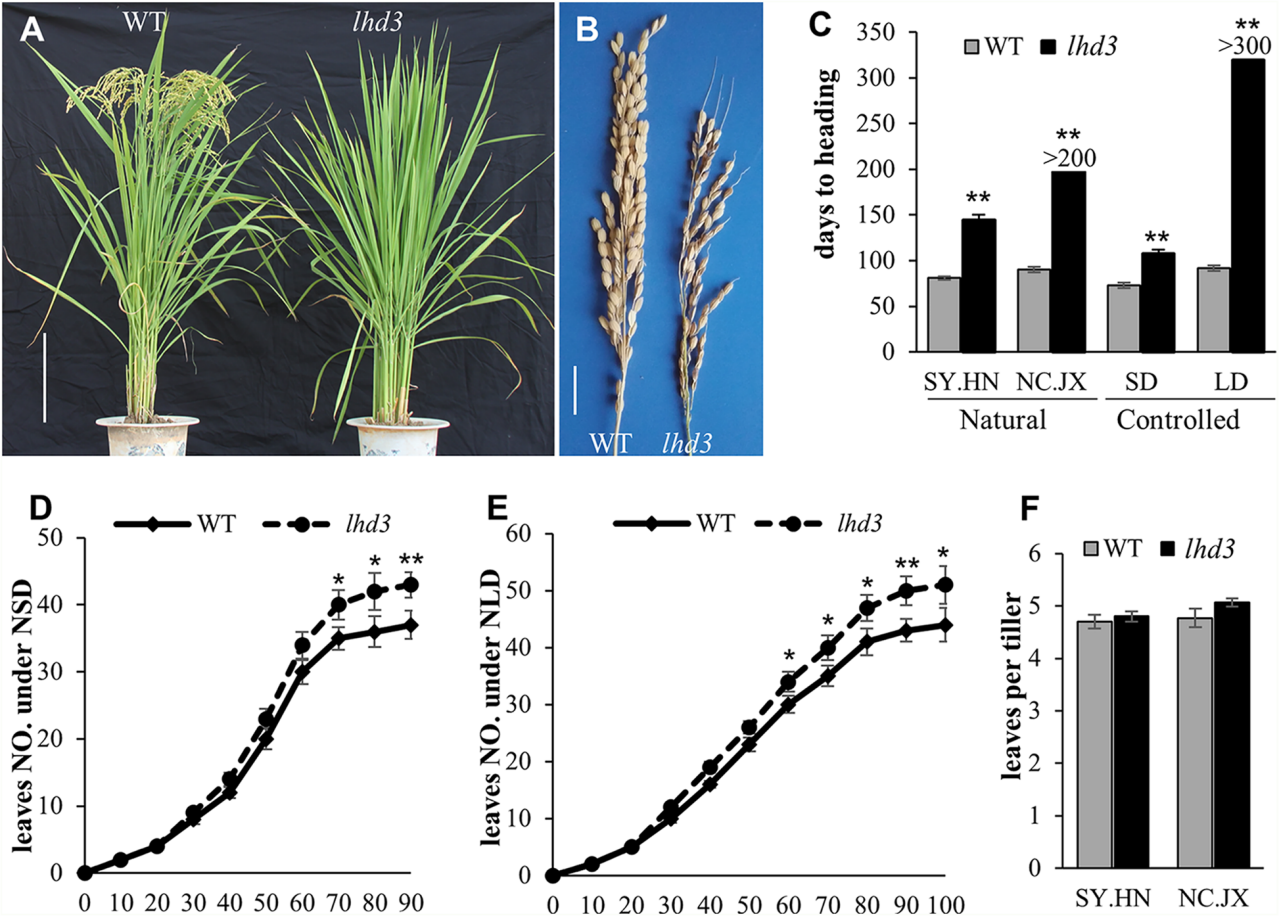
Phenotype comparison of *lhd3* and WT NPB. **A** The phenotype of the WT NPB (left) and the *lhd3* mutant (right) at the mature stage (the plants were cultivated at JXAU in Nanchang city, Jiangxi Province, in 2016). Scale bar = 20 cm. **B** The panicle of WT (left) and *lhd3* (right) in Sanya city, Hainan Province. Scale bar = 2 cm. **C** Days to heading comparison between the WT and the mutant in Nanchang city, Jiangxi Province (NC.JX), Sanya city, Hainan Province (SY. HN) and under controlled short-day (SD) and long-day (LD) conditions. Error bars indicate SD; *n* = 20 plants. **D** and **E** Comparison of leaf emergence rates between the *lhd3* and the WT under natural SD conditions (**D**) and natural LD conditions (**E**). Error bars indicate SD; *n* = 8 plants. **F** Comparison of leaf number per tiller under natural SD conditions (SY. HN) and natural LD conditions (NC. JX). Error bars indicate SD; *n* = 8 plants. * and ** represented the significant difference (*P* < 0.05) and extremely significant difference (*P* < 0.01), respectively

Compared to the WT *japonica cv.* Nipponbare (NPB) plants, the *lhd3* mutant showed a delayed flowering phenotype when grown in both Nanchang, Jiangxi Province (NC.JX, Fig. 1A, B) and Sanya, Hainan Province (SY.HN), China. Under natural SD conditions (SY.HN), the *lhd3* plants flowered on the 148th day after sowing, which was a delay of 64 days compared with the WT NPB plants. When plants were sowed in late May in NC.JX (natural LD conditions), NPB flowered on the 76th day, while *lhd3* plants did not flower even as late as in December. The heading date under controlled LD and SD conditions were consistent with natural conditions. *lhd3* flowered 35 days after WT in 10 h light/14 h dark conditions, but it did not flower until more than 300 days had passed after germinating under 14 h light/10 h dark conditions. The heading time in both the WT and *lhd3* mutant were regulated by photoperiod and positively related to light duration (Fig. 1C). The delay in flowering in the mutant was also in direct proportion to the duration of light. NPB flowered on the 73th, 84th, 76th, and 92th days after sowing in controlled SD, natural SD, natural LD, and controlled LD conditions, respectively; the *lhd3* flowering was delayed by 35 days and 64 days under controlled SD and natural SD conditions, respectively. However, the mutants never flowered under either natural or controlled LD conditions. These results suggest that *LHD3* regulates the flowering of rice via the photoperiod pathway.

To examine whether the delayed flowering in *lhd3* plants was caused by a reduction in growth rate, we counted the leaf emergence rate under both natural LD and SD conditions. Under both photoperiodic conditions, *lhd3* produced more leaves than WT plants at the timepoint that WT NPB flowered. WT plants produced 43 leaves under LD conditions and 36 leaves under SD conditions, while the mutant plants had 50 and 42 leaves under LD and SD conditions, respectively (Fig. 1D and E). The greater number of leaves in the mutant was a result of the greater number of tillers in the mutant plants, and there was no significant difference in the leaf count per tiller between NPB and the mutant (Fig. 1F). The WT produced 4.4 leaves per tiller and the mutant 4.7 leaves per tiller. These results demonstrated that the delayed flowering of *lhd3* under both LD and SD conditions was caused by its prolonged floral transition, not delayed growth.

### Map-based cloning of *LHD3*

**Fig. 2:**
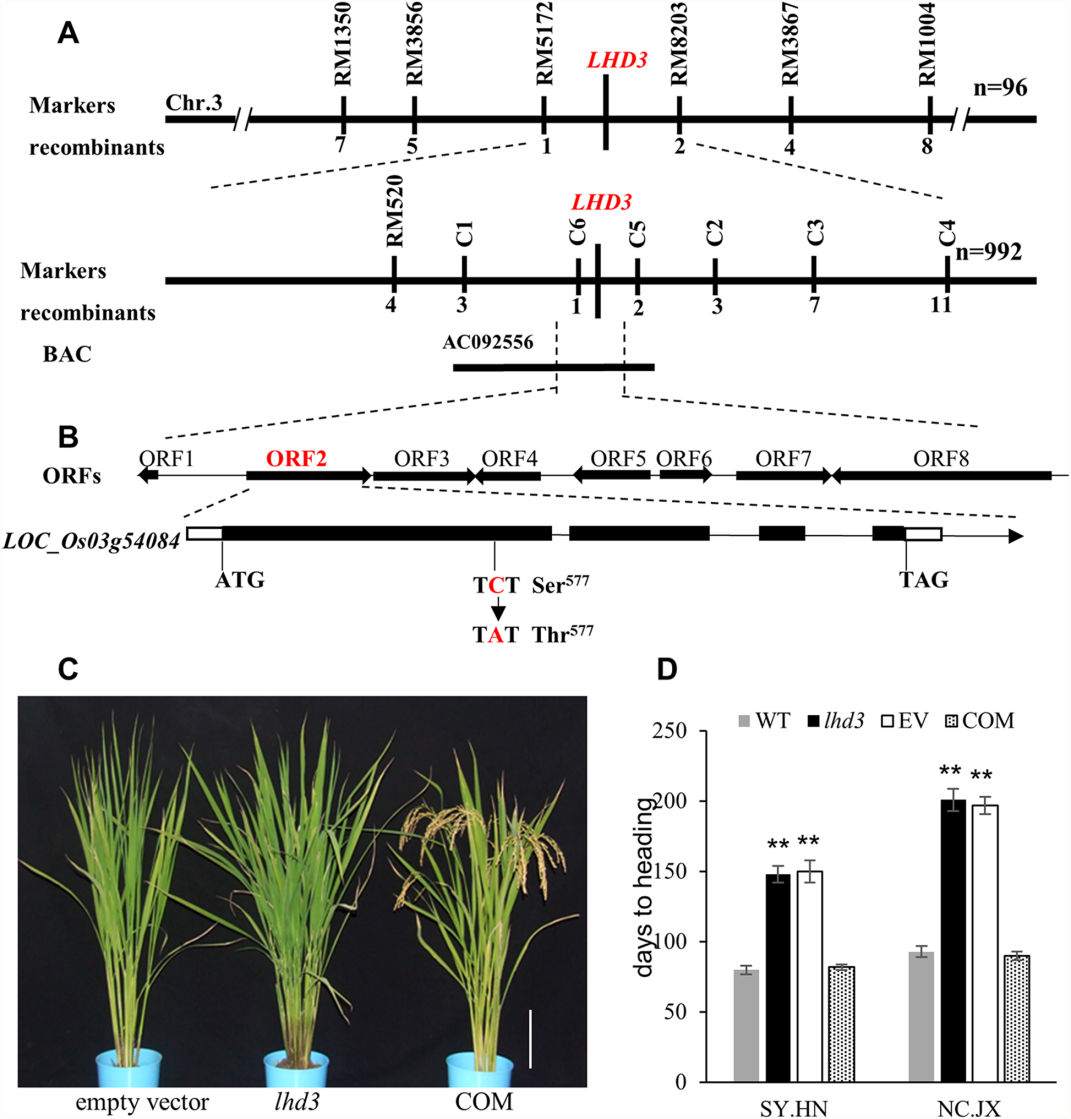
Map-based cloning and identification of the *LHD3* gene. **A** Location of the *LHD3* on the long arm of rice chromosome 3, and the gene was further mapped to a 46 kb interval between molecular markers C5 and C6. **B** The candidate region contained eight ORFs. The mutation site in the *lhd3* was shown at position 1930 in LOC_Os03g54084 and change the serine to threonine at position 577. **C** Phenotypes of transgenic lines. Empty vector was used in the transgenic line as the negative control. Scale bar = 20 cm. **D** Days to heading comparison among the WT, *lhd3* and transgenic plants in both Nanchang city, Jiangxi Province (NC.JX) and Sanya city, Hainan Province (SY. HN). Error bars indicate SD; n = 20 plants. * and ** represented the significant difference (*P* < 0.05) and extremely significant difference (*P* < 0.01), respectively

The *lhd3* mutant was crossed with the WT to ascertain the heredity pattern for the late-heading phenotype. The late-heading phenotype of F_1_ plants and the ratio of individuals of F_2_ (normal : late ≈ 3:1, Table S4) indicated that *lhd3* was controlled by a single recessive gene. Reciprocal crosses between *lhd3* and other *japonica* varieties (i.e., ZH11 and WYJ7) were performed to confirm the segregation ratio, and the results showed a good fit to 3:1 (Table S4). The F_2_ population of *lhd3*/TN1 was employed for isolation of *LHD3* using the map-based cloning strategy. To avoid genetic background diversity effects, 992 non-flowering plants were selected from 8754 F_2_ plants in NC.JX and used for mapping. The mutated locus was roughly mapped to chromosome 3, between RM5172 and RM8203 (Fig. 2A). A large number of sequence-tagged sites (STS) markers were developed, and the location of the *LHD3* locus was narrowed down to an interval of 46-kb region between the two markers C6 and C5 (Fig. 2A), which included eight predicted open reading frames (Fig. 2B). Then all eight genes (containing promoter and coding regions) in both WT and *lhd3* were sequenced. DNA sequencing analysis indicated that only the predicted *LOC_Os03g54084* gene contained a single-base substitution, while the other seven genes did not differ between WT and *lhd3*. A comparison of the *LOC_Os03g54084* CDS from the *lhd3* and WT plants showed that the mutation in *lhd3* resulted in a C to A substitution (1730 C→A) in the first exon, causing an amino acid residue change from serine to threonine (Fig. 2B). According to the *LOC_Os03g54084* gene encoding for phytochrome C in rice, either *LHD3* or *OsphyC* will be used as the gene name in the remainder of this paper.

Molecular phylogenetic analyses indicated that several regions are conserved among phytochromes, and even different classes of phytochromes (Fig. S1), suggesting that these amino acid residues serve common important functions in phytochromes of plants. The mutation site of *lhd3* was located in the PHY domain, and the site was conserved among all phytochromes detected in this study, indicating this serine residue is irreplaceable. Furthermore, the late-heading phenotype of *lhd3* plants was completely restored after the complementation vector (COM) was introduced (Fig. 2C), and the complementation plants flowered on the 86th day and 78th days under natural SD and LD conditions, similarly to the WT (Fig. 2D). These results confirmed that the *OsphyC* was responsible for the *lhd3* phenotype.

### OsphyC is required for light-dependent Chl synthesis

**Fig. 3:**
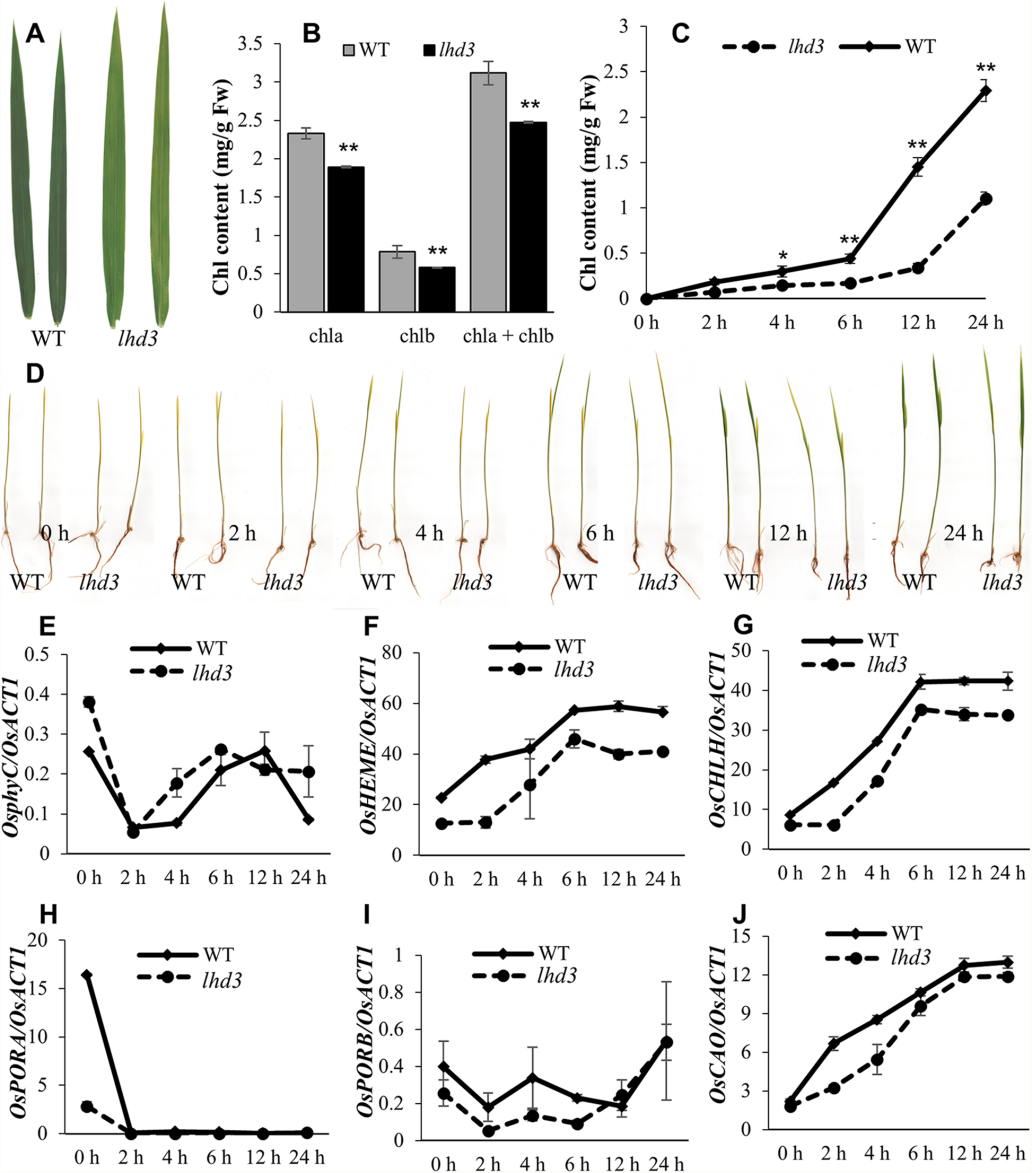
Chl synthesis and expreesion of related gene expression in the WT and the *lhd3. ***A** Leaf color of the WT (left) and the lhd3 (right) at the tillering stage. **B** Chl content of the WT and the lhd3 at the tillering stage. **C** Chl content in the WT and the lhd3 seedlings during greening. **D** Comparison of greening rate in the WT and the lhd3 etiolated seedlings that were exposed to light (200 µmol m^− 2^s^− 1^) for 0–24 h. **E**–**J** Changes in transcript levels of LHD3 (**E**) and Chl synthesis-associated genes in WT and pgl seedlings during greening, including OsHEME (**F**), OsCHLH (**G**), OsPORA (**H**), OsPORB (**I**), OsCAO (**J**). Mean and SD values in Chl content and qPCR analysis were obtained in each experiment with three biological replicates, and tissues from more than 10 plants were used for every experiment to avoid individual difference. * and ** represented the significant difference (*P* < 0.05) and extremely significant difference (*P* < 0.01), respectively

The *lhd3* mutant exhibited a moderate pale-green leaf phenotype in the paddy field (Fig. 3A), and its Chl contents were lower than those of the WT (Fig. 3B), suggesting that OsphyC is involved in Chl synthesis or degradation. To further examine the role of OsphyC in seedling greening, we exposed dark-grown seedlings to continuous white light. The etiolated WT seedlings quickly turned green after being exposed to light, while most of the *lhd3* seedlings remained pale green after 24 h of light exposure (Fig. 3D). Compared with etiolated WT seedlings, *lhd3* seedlings had significantly slower Chl synthesis levels. After 24 h of exposure, the total Chl content reached 2.3 mg/g FW (fresh weight) in the WT which doubled that in mutants (Fig. 3C). These results suggest that OsphyC plays an important role in the light-dependent accumulation of Chl.

Subsequently, the expression levels of several Chl-synthesis-associated genes and *OsphyC* during greening were detected. Interestingly, the expression pattern of *OsphyC* was found to be complex. In the WT plants, *OsphyC* was highly expressed in the dark, and was down-regulated under continuous light exposure. A similar expression pattern of *OsphyC* was obtained in the mutant, in which *OsphyC* expression was marginally higher than that in the WT in both dark and light conditions (Fig. 3E), suggesting that the pale green leaf phenotype of *lhd3* plants was caused by a dysfunction of OsphyC protein rather than its transcriptional level. The transcript levels of *OsCAO*, *OsHEMA*, *OsCHLH*, and *OsPORB* were increased in both WT and *lhd3* plants after illumination (Fig. 3F, G, I, J) and were lower in the mutant than WT plants. These results suggest that OsphyC is essential for the light-dependent accumulation of high levels of Chl in rice, and acts via regulating key genes involved in Chl synthesis. Interestingly, OsPORA, a light-inhibited and specifically darkness-expressed protochlorophyllide oxidoreductase, was also drastically down-regulated in *lhd3*, even under dark conditions (Fig. 3H). These results suggest that OsphyC is involved in both light-dependent and light-independent pathways.

### Effects of OsphyC on photoperiod pathway gene expression

**Fig. 4:**
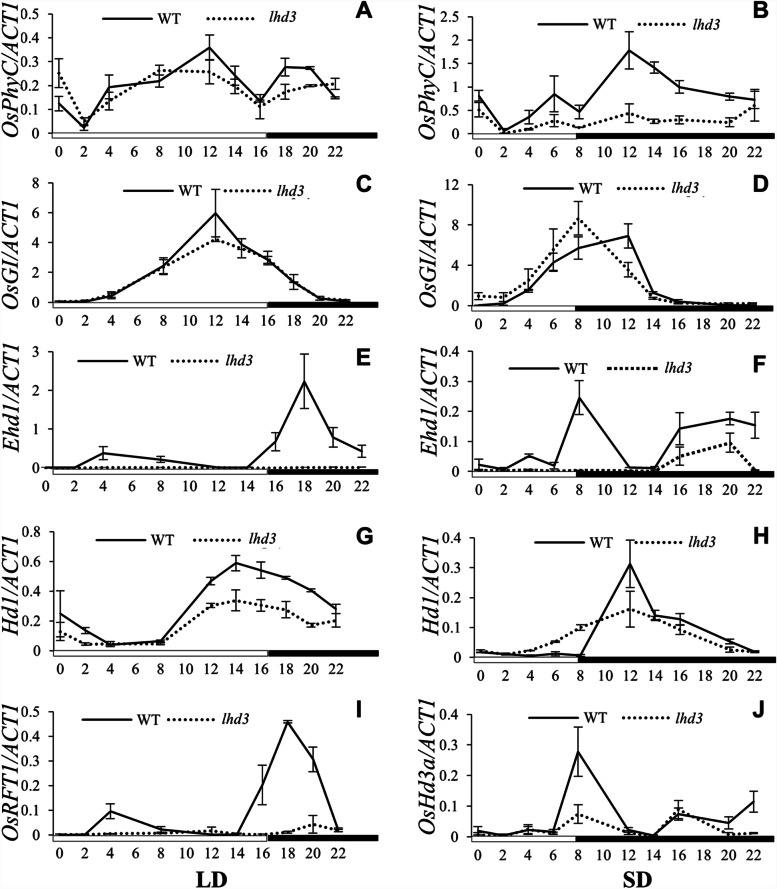
Diurnal expression patterns of flowering regulators in the WT and the *lhd3. *Diurnal expression patterns of OsphyC (**A** and **B**), OsGI (**C** and **D**), Ehd1 (**E** and **F**), Hd1 (**G** and **H**), OsRFT1 (**I**), and OsHd3a (**J**) in the WT and the lhd3 under controlled LD and SD conditions. The expression levels of Actin gene was used as a control. In all panels, the mean of each point is based on the average of three biological repeats calculated using the relative quantification method, tissues from more than 10 plants were used for every experiment to avoid individual difference

Flowering was delayed in *lhd3* under both LD and SD conditions. Thus, the mRNA levels of flowering-time genes (*OsGI*, *Ehd1*, *Hd1*, *OsHd3a*, and *OsRFT1*) were examined under both LD and SD conditions. In WT plants, *OsphyC* was determined to be expressed at low levels under LD conditions and at higher levels under SD conditions. Whereas *OsphyC* transcript levels were low in both conditions in the mutant. Thus, *OsphyC* was expressed similarly in WT and *lhd3* under LD but at lower levels in the mutant compared with WT under SD (Fig. 4A, B). OsphyC had no effect on the expression of *OsGI*, which showed a similar expression pattern in *lhd3* and WT under both LD and SD conditions (Fig. 4C, D). *Hd1* was mildly affected by OsphyC, and the OsphyC mutation suppressed the expression of *Hd1* under both conditions, although *Hd1* was less affected under SD (Fig. 4G, H). The expression of *Ehd1* was sharply suppressed in *lhd3* under both conditions, especially under LD. *Ehd1* was hardly detected, while a very low levels of *Ehd1* transcripts were detected under SD (Fig. 4E, F). Both florigens involved in SD and LD (OsHd3a and OsRFT1, respectively) were down-regulated in *lhd3*. *OsRFT1* expression was nearly completely suppressed under LD (Fig. 4I); the suppression of *OsHd3a* under SD conditions was somewhat less severe than that of *OsRFT1* under LD conditions (Fig. 4J), as the latter resulted in a complete absence of flowering under LD, while there was only a 1-month-delay in heading under SD conditions in *lhd3* plants. Taken together, OsphyC functions upstream of Ehd1, Hd1, OsHd3a and OsRFT1 in the photoperiodic flowering pathway, and OsphyC might function in both Hd1-dependent and Ehd1-dependent pathways in rice.

### Disrupted dimerization of OsphyC with itself or OsphyB in *lhd3*

**Fig. 5:**
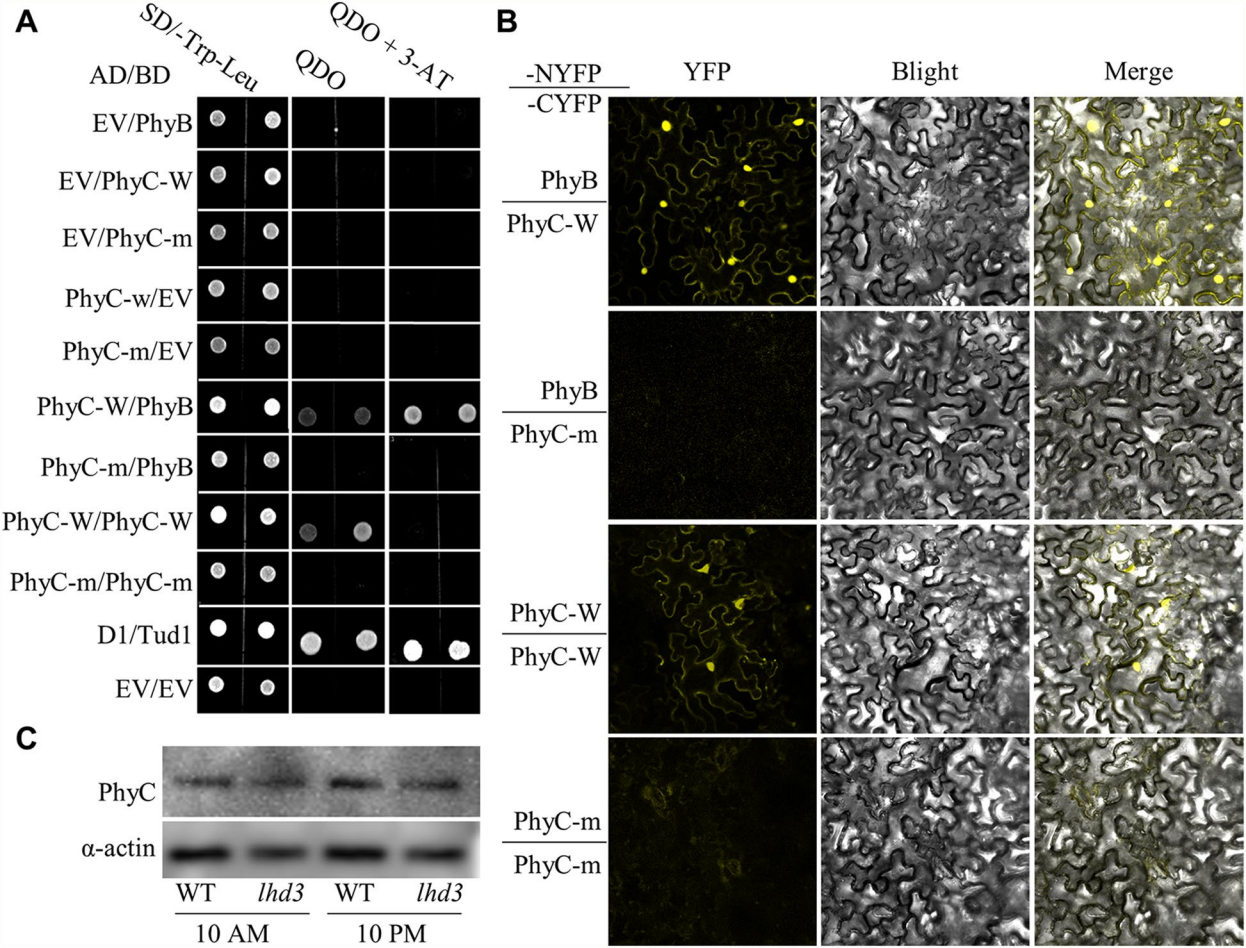
Disrupted dimerization of OsphyC in the *lhd3*. **A** Yeast two-hybrid assay of interactions between OsphyC and OsphyB (or OsphyC) from the WT and the lhd3. The interaction between D1 and TUD1 was used as a positive control, and empty pGBKT (bait) and pGADT (prey) were used as negative controls. QDO indicates SD/-Trp-Leu-His-Ade medium. **B** BiFC assay of the interaction between OsphyC and OsphyB (or OsphyC) from the WT and the lhd3 in N. benthamiana epidermal cells. **C** OsphyC protein levels at two selected time points (10 AM and 10 PM) in the WT and the lhd3 plants grown under natural LD conditions. Each lane was loaded equally, and followed by protein gel blot analyses using antibodies against OsphyC and OsActin as the loading control

Previous studies showed that PhyC interact with PhyB to form heterodimer [9, 19]. The outcomes of a yeast two-hybrid (Y2H) assay implied that OsphyB, normal OsphyC (OsphyC-w), and mutational OsphyC (OsphyC-m) have no transcriptional activation activity (Fig. 5A); OsphyC interacted with OsphyB, forming heterodimer, in WT plants, while this interaction was weakened or disrupted in *lhd3*. Interestingly, we observed a relatively weak interaction between OsphyC-w and itself, which has never before been reported. Yeast cells co-transformed with plasmids expressing OsphyC-w respectively fused with AD and BD were successfully grown on selection medium with no 3-AT, whereas there was a lack of growth on selection medium ( SD/-Ade/-His/-Trp/-Leu) with with 5 mM 3-AT (Fig. 5A). Similar to the disruption of OsphyC-m’s ability to form heterodimers, OsphyC-m was also unable to form a homodimer (Fig. 5A). A bimolecular fluorescence complementation assay (BiFC) showed consistent results. Strong fluorescence signals of yellow fluorescent protein (YFP) were observed in the cytoplasm and nuclei of leaf pavement cells when OsphyC-w-NYFP and CYFP-OsphyB were co-expressed, while no fluorescence signal was observed when the mutant fusion protein OsphyC-m-NYFP was expressed (Fig. 5B). A similar experiment was conducted to verify the formation of OsphyC homodimers. Pavement cells with OsphyC-w-NYFP and CYFP-OsphyC-w showed active fluorescence signals of YFP, but those with OsphyC-m-NYFP and CYFP-OsphyC-w did not (Fig. 5B). These results implied that the substituted amino acid in OsphyC-m is essential for dimerization, both the heterodimerization of OsphyB/OsphyC and homodimerization of OsphyC/OsphyC.

It has been reported that the stability of PhyC is related to dimerization of PhyB/PhyC in both *Arabidopsis* and rice [11, 20]. OsphyC levels in the plants were detected by its antibody. Under both light and dark conditions, OsphyC levels were lower in the *lhd3* mutant; in terms of increasing accumulation levels, the order was WT in darkness, WT in light (slightly less than that in darkness), *lhd3* in darkness, and *lhd3* in light (Fig. 5C, Fig. S2 and Fig. S3), suggesting that the disruption of OsphyC dimerization caused by a single-nucleotide polymorphism in PHY domain also affected OsphyC stability.

## Discussion

### PHY domain is important for phytochrome dimerization

Plant phytochrome genes are very conserved among species. As shown in Fig. S1, besides AtPhyC, AtPhyA and AtPhyB are also highly homologous with PhyC proteins. Phytochromes are composed of a conserved domain structure, PLD-GAF-PHY-PAS-PAS-HKRD, in which the N-terminal chromophore-binding domain has been widely believed to function as the photosensing domain, and the C-terminal is considered to be involved in signal transmission [21, 22]. Phy dimerization is a common occurrence in plants and is important for the functioning of the light signal transduction pathway. PAS domains are believed to be essential for phytochrome dimerization [23]. A series of experiments, including analysis of the behaviors of phytochrome fragments expressed in vivo, interaction assays in yeast and bacterial, and site-directed mutagenesis, indicated that several regions at the C-end function in phytochrome dimerization. Yamamoto and Tokutomi obtained a dimeric aa 753–1089 fragment of pea PhyA, suggesting that this region contains the domain for dimerization [24]. Using an *Escherichia coli* in vivo two-hybrid assay, the aa 623–673 region was found to be required for oat phyA dimerization [25]. Subsequently, it was confirmed that the aa 599–683 region containing aa 623–673 was necessary but insufficient for dimerization. However, neither the following PAS2 domain nor the previously reported functional aa region 1069–1129 was required [26]. Moreover, loss of the aa 652–712 region in PhyB did not affect dimerization when expressed in *Arabidopsis* [27], suggesting that it is not involved in the dimerization of native phytochromes. The aa 919–1093 region was demonstrated to be necessary for PhyA dimerization in tobacco using transgenic plants with various truncations in the C-terminal domain [28]. Although several different regions within the C-terminal, including PAS1, PAS2, and HKRD domains, were implied to be responsible for Phy dimerization [22], the exact dimerization domain is still controversial.

Compared with PhyA and PhyB, PhyC is rarely concerned, and its functions and structure are unclear. Observations of the heterodimerization of OsPhyC and OsPhyB in rice were mentioned in a previous report, but the domains required for dimerization were not discussed [19]. Based on the results of the current study, we propose that the 577th serine is important for the dimerization of OsPhyC. Interestingly, this serine residue is located in the PHY domain within the N-terminal, a domain that has never been considered to mediate dimerization. The 577th serine is greatly conserved in every Phy of all species (Fig. S1), indicating that this amino acid is very important to Phy, and it may be conserved in all Phys. The PHY domain is adjacent to the PAS domains. Amino acid mutations in PHY might cause conformation transitions that easily result in dysfunction of the PAS domains, including disable dimerization. However, although many studies have focused on Phy dimerization, no evidence has emerged showing the PHY domain functions in these interactions. Most researchers have assumed the domain required for dimerization possibly exists in the C-end and, thus, have carried out dimerization analyses using internal deletion or site-specific mutation almost entirely targeting the C-terminal of Phy, while the domains in the N-terminal (especially the PHY domain) were always ignored in their experimental design. Thus, they revealed that the PAS2 domain is required for dimerization of Phys which contained functional PHY domains [22, 25, 27, 28]. However, many results indicate that several domains in the C-end are required but not sufficient for dimerization [25, 26]. Thus, there are some other domains, such as the 577th serine within PHY domain, might be also important for Phy dimerization.

### OsphyC regulates photoperiodic flowering in rice

Light is known to be important for heading in rice [29], and unsurprisingly, phytochromes are involved in this process. Various combinations of single- and double- phytochrome mutants have different heading dates, suggesting that OsphyA, OsphyB, and OsphyC have different effects on flowering [11]. A similar flowering time was observed in *osphyC* and *osphyB* under LD: *osphyB* flowered earlier under SD conditions, while *osphyC* flowered at the same time as WT. The flowering time of *osphyB/osphyC* double mutants was the same as that of *osphyB* monogenic mutants under both LD and SD conditions [11]. These results implied that OsphyC and OsphyB have redundant functions in photoperiod flowering, and the function of OsphyC is OsphyB-dependent. Furthermore, The heterodimer of OsPhyB/OsPhyC is responsible for stabilizing OsPhyC. OsPhyC levels were shown to be decreased in etiolated seedlings of *osphyB* mutants but were recovered by an inactive form of chromophore-less OsPhyB (C364A), which was able to interact with PhyC. These results suggested that PhyC was stable in heterodimer form in etiolated seedlings, even when PhyB is inactive [19]. In *lhd3*, the interaction between OsphyC and OsphyB was weakened, resulting in lower OsphyC levels, which might have been responsible for the delayed heading.

There exists both an evolutionarily conserved pathway (OsGI-Hd1-OsHd3a) and a unique pathway (Ghd7-Ehd1-OsHd3a/OsRFT1) for the photoperiodic control of flowering in rice when compared to *Arabidopsis* [30]. OsphyB is involved in the repression of *OsHd3a* by *Hd1* under LD conditions, and *osphyB* mutants attenuate the conversion of the activator to the suppressor and maintain Hd1 as an activator under both photoperiodic conditions [31–33]. OsphyB also suppress *Ehd1* expression via *OsCOL4*, a CONSTANS-like gene [31, 34]. In *lhd3*, *OsGI* was hardly affected (Fig. 4C, D), *Hd1* was mildly suppressed (Fig. 4G, H), and *Ehd1* was sharply suppressed under the both light conditions (Fig. 4E, F). Moreover, *Ehd1* and florigin genes were less affected under SD compared with under LD conditions, which is consistent with the flowering phenotype of *lhd3*. Therefore, OsphyC may function in photoperiodic flowering mainly via the regulation of an Ehd1-dependent pathway.

However, the functions of PhyC in flowering regulation remain enigmatic. The only earlier-reported *osphyC* mutant showed early heading under LD conditions and no significant phenotype under SD conditions in Takano et al.’s study, but its heading date was not significantly altered under either LD or SD conditions in Osugi et al.’s experiments [11, 13]. The rice *osphyC* mutant *lhd3* featured in this study had totally different, even completely opposing, heading phenotypes, i.e., delayed flowering time under both LD and SD conditions. It is possible that the different mutation sites between in the earlier-reported *osphyC* and *lhd3* cause contrary flowering-phenotype. Similar conditions were found in some other genes. For instance, a novel mutant of *d11* (*sg4*) was higher than the wild type, different from other typical *d11* mutants with dwarfing phenotype [35, 36]; The substitution from Arg to Ser at position 163 of CLG1 that enhances the E3 ligase activity of CLG1 and thus increases rice grain size, while overexpression of mutated *CLG1* with changes in three conserved amino acids decreased grain length [37]. Moreover, *phyC* mutants of different species may display contrary flowering phenotypes [15, 17, 18]. Interestingly, the reported *osphyC* mutant showed a similar heading phenotype to *Arabidopsis atphyC* mutants, while *lhd3* was similar to several monocot *PhyC* mutants, such as wheat and *Brachypodium distachyon*. Thus, more detail work will be needed to fully understand how OsphyC regulates heading in rice.

## Conclusion

In this study, we identified a novel *osphyC* mutant, *lhd3*. Amino acid residue change from serine to threonine at the 577th residue of OsphyC led to pale green leaves and delayed flowering time in the *lhd3*. Moreover, the 577th serine in the PHY domain is essential for OsphyC dimerization that is important for its functions in photomorphogenesis, including Chl synthesis and photoperiodic flowering. Our results will facilitate efforts to further elucidate the functions and mechanism of OsphyC during light signal transduction in rice.

## Materials and methods

### Plant materials and growth conditions

The *lhd3* mutant was isolated from a mutant population of the NPB, which was created by ethyl methane sulphonate (EMS) mutagenesis. The *lhd3* was crossed with the WT NPB and several *japonica* varieties for genetic analysis and with the typical *indica* cv TN1 for mapping population construction. To observe the plants under natural conditions, they were grown in a paddy fields in Nanchang (E28.77, N115.84), Jiangxi Province, China, for LD conditions and Sanya (E109.51, N18.254), Hainai Province, China, for SD conditions. For phenotypic analysis under controlled photoperiod treatment, the plants were grown in growth chambers with 14 h light/10 h dark for LD conditions, while 10 h light/14 h dark for SD conditions. For photoperiod pathway gene expression analysis, seedlings of the *lhd3* mutants and WT were grown for 30 days under natural daylength conditions, then transferred to chambers for treatment under LD (16 h light/8 h dark) and SD (8 h light/16 h dark). After being entrained for 5 days, leaves were harvested to extract total RNA for qPCR.

### Map-based cloning of *LHD3*

The F_2_ population from the crossing of *lhd3* and TN1 was used for gene mapping. As heading-time is a quantitative character, extremely delayed flowering F_2_ individuals were selected for gene cloning. Firstly, a DNA bulk pool from 33 F_2_ mutant individuals was used for preliminary linkage analysis and screened with a total of 224 simple sequence repeat (SSR) markers scattered among all 12 chromosomes (www.gramene.org). Subsequently, 96 individuals were used for primary gene mapping. For fine mapping, STS markers were developed between the two flanking markers based on comparisons between the genomic DNA sequences of NPB and the *indica* cultivar 9311 (www.gramene.org/resources). PCR products were separated on 4–5% agarose gels or 8–12% polyacrylamide gels. The sequences of the primers used for mapping are shown in Table S1.

### Complementation test

A 7638-bp genomic DNA fragment (containing the entire *LHD3* coding region, a 2253-bp upstream region, and a 916-bp downstream sequence of *LHD3*) was amplified from the WT and inserted into the binary vector pCAMBIA1300 to generate the transformation vector pLHDF (COM). The binary construct was introduced into calli generated from the mature seed embryos of *lhd3* using an *Agrobacterium* (EHA105)-mediated method. Rice transformation was performed as previously described [38]. The sequences of the primers used for vector construction are shown in Supplementary Table 2 .

### Chl content determination

The total Chl content in the leaves and seedlings was extracted with 80% acetone, and analyzed with a spectrophotometer (Shimadzu UV2400, Japan). The total Chl, Chla, and Chlb contents were estimated with spectrophotometric values of 470 nm, 645 nm, and 663 nm, using methods described in a previous report [39].

### RNA extraction and quantitative real-time PCR (qPCR)

Total RNA was extracted from different tissues of rice plants using the RNeasy Plant Mini Kit (Qiagen), cDNA was synthesized by reverse transcription using the PrimeScript II 1st Strand cDNA Synthesis Kit (Takara), and genomic DNA was digested using DNase I (Takara). qPCR was carried out with 2×SYBR Green PCR Master Mix (Applied Biosystems) in an ABI 7900HT Real-Time PCR System. Several genes involved in Chl synthesis and flowering were quantified: *OsCAO1*, *OsPORA*, *OsPORB*, *OsHEMA*, *OsCHLH*, *OsGI*, *Ehd1*, *Hd1*, *OsHd3a* and *OsRFT1*. *OsACTIN1* was used as an internal control. At least three biological replicates were performed for each experiment. The sequences of the primers used in the qPCR are shown in Table S3.

### Yeast two-hybrid (Y2H) assay

The full-length *OsphyB*, *OsphyC-w* (the full WT NPB OsphyC gene) and *OsphyC-m* (the mutational *OsphyC* allele in the *lhd3* mutant) genes were cloned into either a pGBKT7 or pGADT7 vector at the *Eco*R I site with ClonExpress II One Step Cloning Kit (Vazyme). Then different combinations were co-transformed into AH109 cells for testing transactivation activity, using pGBKT7/pGADT7 for the negative control and BD-OsTub1/AD-D1 for the positive control. Yeast clones were grown on SD/-Trp/-Leu or SD/-Ade/-His/-Trp/-Leu medium (selection medium) for 3–4 days at 30 ℃. 5 mM 3-AT (3-amino-1,2,4-triazole) was added in the selection medium to detect the strength of protein interaction. Positive clones were tested for the presence of appropriate plasmids using colony PCR. The primers used for Y2H-related vector construction are shown in Table S2.

### Bimolecular fluorescence complementation (BiFC) assays

*OsphyC-w* and *OsphyC-m* were individually inserted into both 35 S::nYFP and 35 S::cYFP vectors containing either N- or C-terminal YFP fragments, and the *OsphyB* CDS was cloned into a 35 S::nYFP plasmid. The recombinant NYFP and CYFP vectors were co-expressed in *Nicotiana benthamiana* pavement cells. *Agrobacterial* infiltration of tobacco leaves was performed as previously described [40]. Leaf samples were taken 48–72 h after agrobacterial infiltration and observed under an Axio Observer inverted microscope equipped with the LSM800 laser scanning confocal module (Carle Zeiss). The specific primers used to produce the BiFC constructs are listed in Supplemental Table S2.

### Western blot analysis

To analyze OsphyC protein levels in plants, leaves from both WT and *lhd3* grounder light and darkness at the tillering stage were harvested, and total proteins were extracted with extraction buffer (20 mM Tris-HCl [pH 7.5], 150mM NaCl, 2.5 mM EDTA, 1% Triton X-100, and 1% β-mercaptoethanol), and the protein concentrations were determined using the Enhanced BCA Protein Assay Kit (Beyotime). The proteins were subsequently separated om 8% SDS-PAGE gels and analyzed using anti-OsphyC and anti-α-actin antibodies (Abmart). To prepare the OsphyC polyclonal antibody, a polypeptide with the sequence AKHEPIDADDNGRK, which is specific to OsphyC was artificially synthesized as an antigen to stimulate an immune response in rabbits.

## Supplementary Information

**Additional file 1.**

## Data Availability

Sequence data can be found at NCBI databases (www.ncbi.nlm.nih.gov): OsphyC (Os03g0752100, XP_015630523.1), OsphyB (Os03g0309200, NP_001389072.1), OsphyA (Os03g0719800, XP_015630340.1), AtphyC (At5g35840, NP_198433.1), AtphyB (At2g18790, NP_179469.1), AtphyA (At1g09570, NP_001322906.1), ZmphyC (XP_008665426.1), TaphyC (XP_044399157.1), and SbphyC (AAR33021.1).

## Abbreviations

Chl: Chlorophyll
EMS: Ethyl methane sulphonate
Pfr: Far red-absorbing form
LHD: Late heading date
Pr: Light-absorbing form
phy: Phytochrome
STS: Sequence tagged site
SSR: Simple sequence repeat
