# Supplementary material for: A base substitution in OsphyC disturbs its Interaction with OsphyB and affects flowering time and chlorophyll synthesis in rice
Source: BMC Plant Biol. 2022 Dec 27;22:612. doi: 10.1186/s12870-022-04011-y (PMC9793604; doi:10.1186/s12870-022-04011-y)
Supplement: Supplementary file 1 — Additional file 1. [file 12870_2022_4011_MOESM1_ESM.doc]

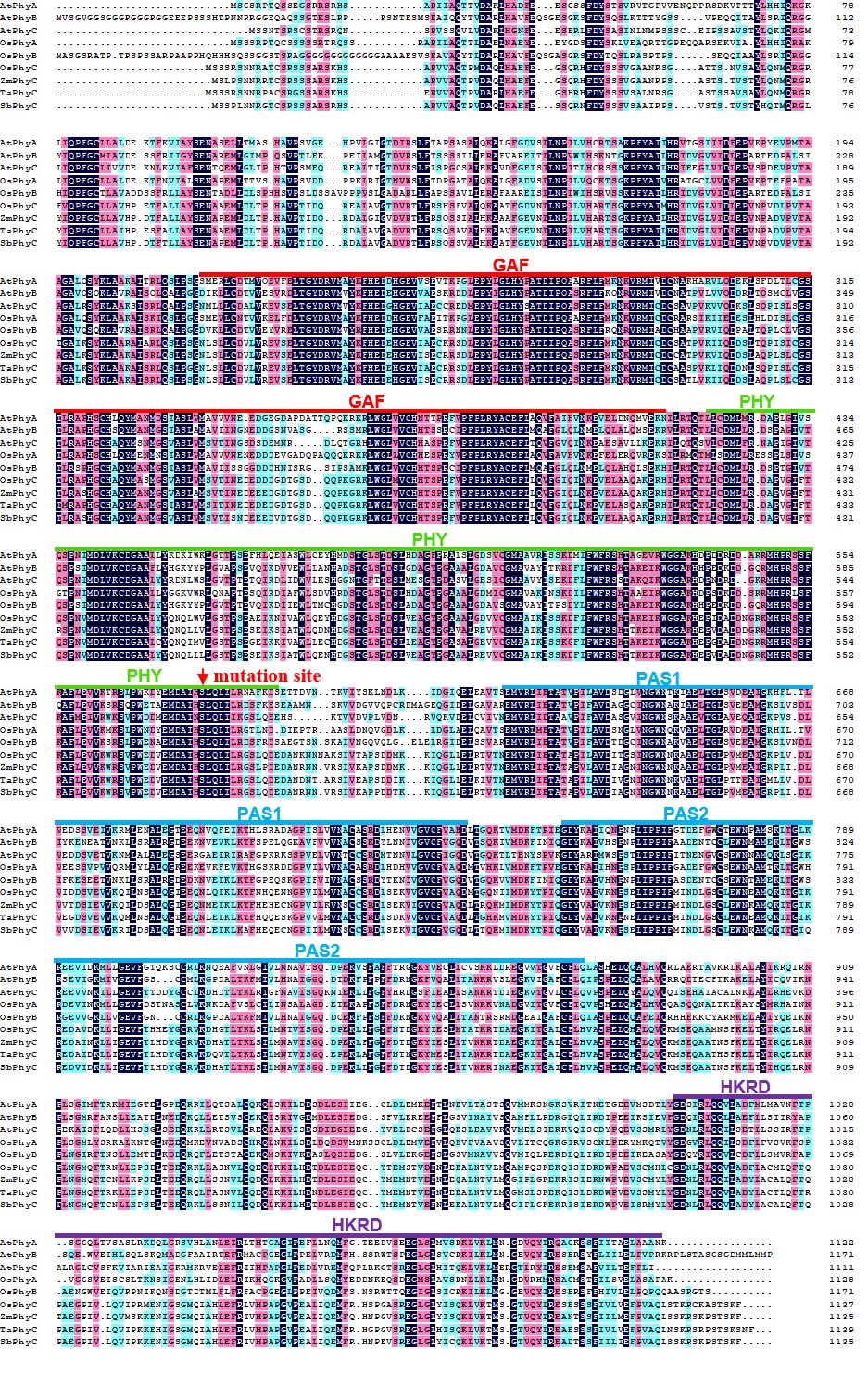


Fig. S1 Molecular phylogenetic analysis of phytochromes in plants.


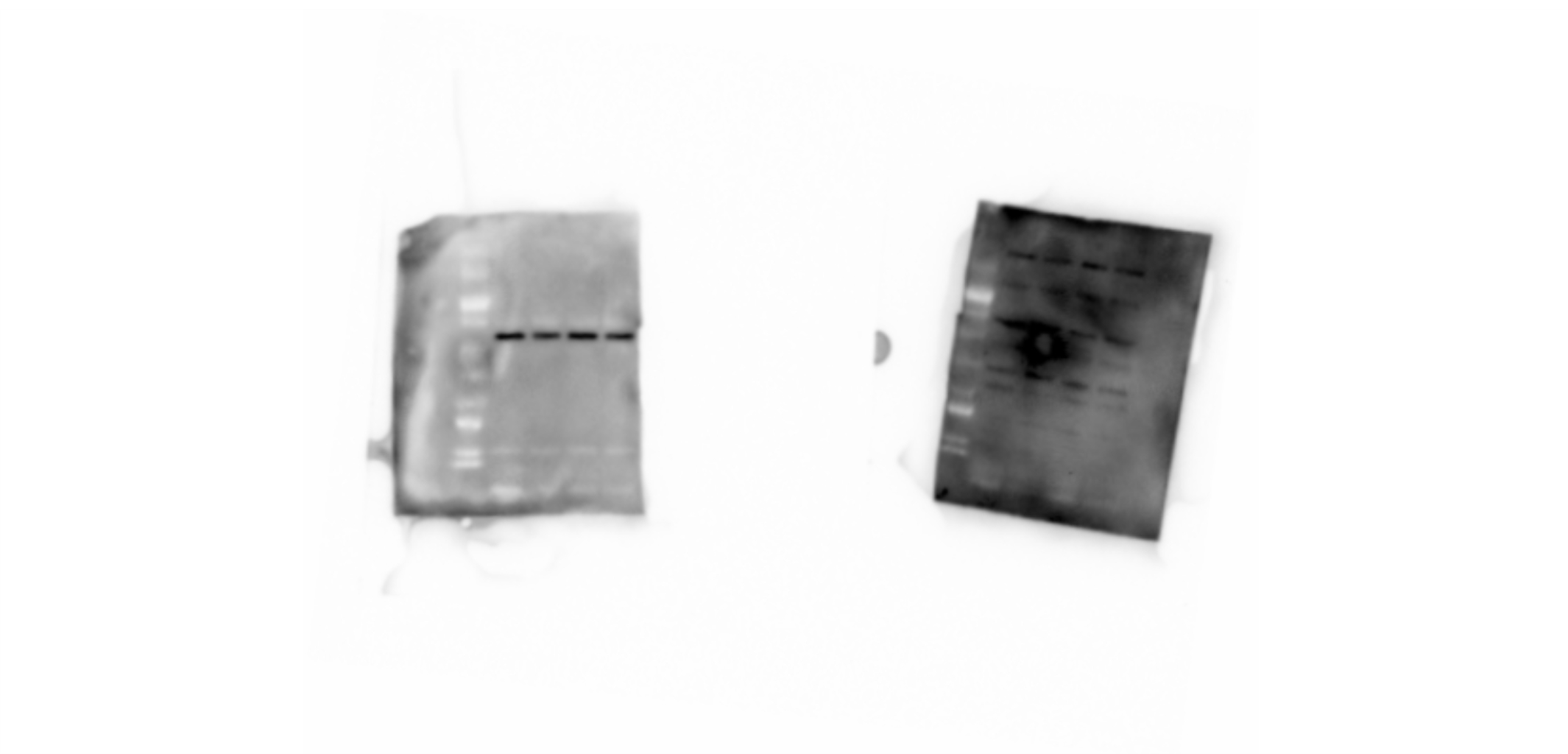


Fig. S2 The original image of western blot assay with anti-α-actin antibody.


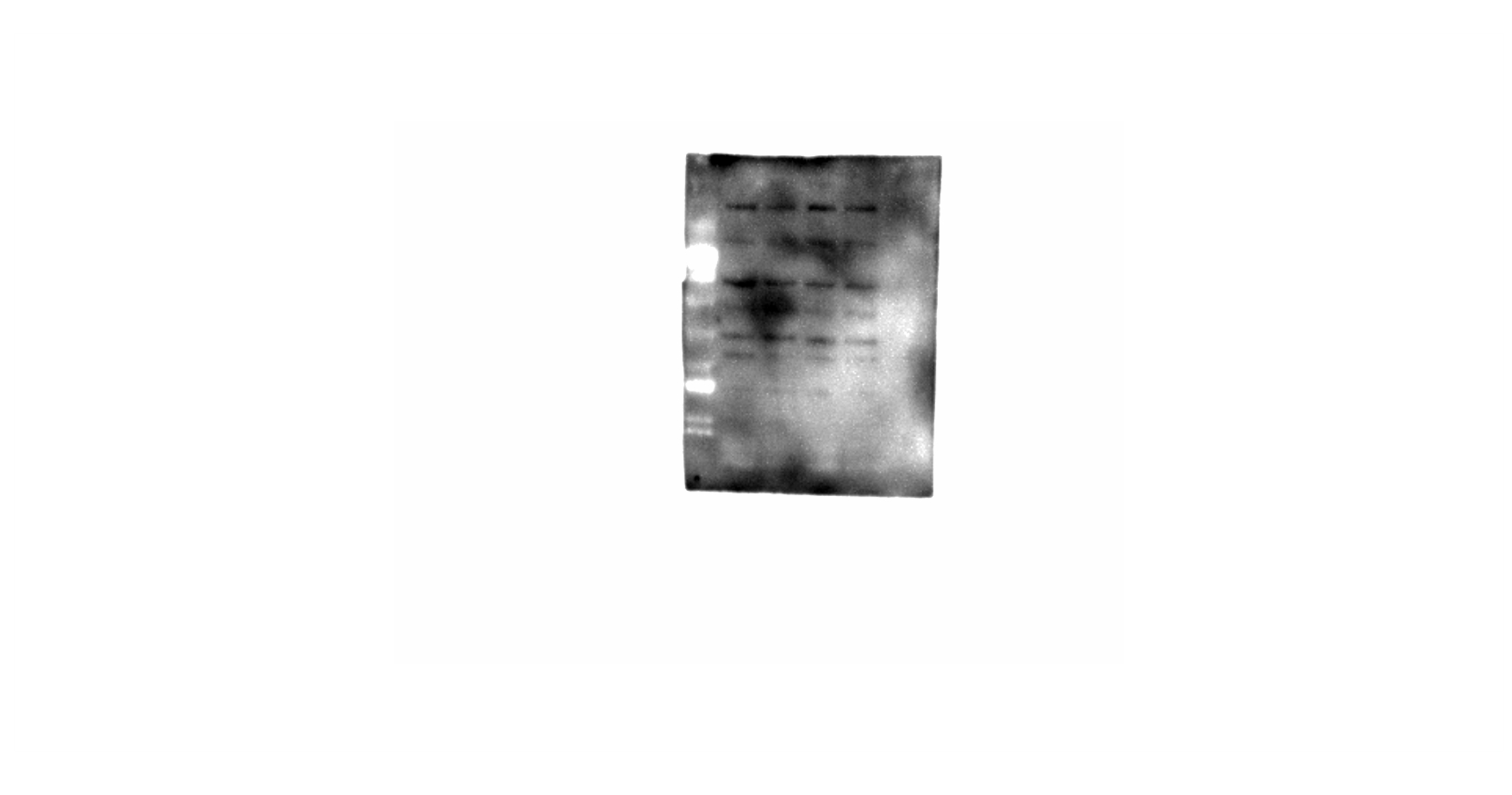


140 KD

95 KD

70 KD

55 KD

43 KD

33 KD

26 KD

20 KD

15 KD

190 KD

Fig. S3 The original image of western blot assay with anti-OsphyC antibody.

Tab.S1 Primers for mapping of *LHD3*

| Mark | Forward Primer | Reverse Primer |
| --- | --- | --- |
| C1 | GTGCTCCGTAGGCTCATCTC | GACGACCCTTTCTGGAACAG |
| C2 | AGCTGATCTGCTCAAGCTGTGG | CAGTCTCTCTCGGCCAATTAAGC |
| C3 | GACTTTGATTAGACAGAGGCCAAGC | GCGCCAGGACTAAACTAAACAGC |
| C4 | ACATTTCGTGCTGAAAGACTGG | GAAATCAGTGTACGTAGGATCAGAG |
| C5 | GTTCTCGATCCGCTTCAGCTGCACC | CGTCGCTGATAGCGAGGTGGGTAGG |
| C6 | AATCCATGTTAACTTATCCTAC | GCCACATAATGGTACTTAATTAGC |

Tab. S2 The sequences of the primers used for vectors construction

| Primers | Sequence | Using for |
| --- | --- | --- |
| COM-Fw | CCATGATTACGAATTCAATTGTGGGTCATGTATTGGAGTCAAAGGC | Complementation test |
| COM-Re | CGACTCTAGAGGATCCGGACGTCGTAGCTGCAGGCGCTGCAAGCGC |
| PhyC-AD-F | GCCATGGAGGCCAGTGAATTCATGTCGTCGTCGCGGTCGAACAACCG | OsPhyC-w and OsPhyC-m inserted to pGADT7 |
| PhyC-AD-R | CAGCTCGAGCTCGATGGATCCTCAGAATTTACTCGTGGAAGCCTTGCAC |
| PhyB-AD-F | GCCATGGAGGCCAGTGAATTCATGGCCTCGGGTAGCCGCGCCACGCCC | OsPhyB inserted to pGADT7 |
| PhyB-AD-R | GCCATGGAGGCCAGTGAATTCTCAGCTTGTCCCCCTACTTGCTGC |
| PhyC-BD-F | ATGGCCATGGAGGCCGAATTCATGTCGTCGTCGCGGTCGAACAACCG | OsPhyC-w and OsPhyC-m inserted to pGBKT7 |
| PhyC-BD-R | CCGCTGCAGGTCGACGGATCCTCAGAATTTACTCGTGGAAGCCTTGCAC |
| PhyB-BD-F | ATGGCCATGGAGGCCGAATTCATGGCCTCGGGTAGCCGCGCCACGCCC | OsPhyB inserted to pGBKT7 |
| PhyB-BD-R | CCGCTGCAGGTCGACGGATCCTCAGCTTGTCCCCCTACTTGCTGC |

Tab. S3 Primers used in qRT-PCR

| Gene | Forward Primer | Reverse Primer |
| --- | --- | --- |
| *OsPhyC* | GTGCTCCAAGTCATCCCCAGGATGG | CTCTCGAGATGCACCTGGGCTGTGG |
| *OsCAO1* | GATCCATACCCGATCGACAT | CGAGAGACATCCGGTAGAGC |
| *OsHEMA* | CGCTATTTCTGATGCTATGGGT | TCTTGGGTGATGATTGTTTGG |
| *OsCHLH* | AACTGGATGAGCCAGAAGAGA | AAATGCAAAAGACTTGCGACT |
| *OsPORA* | ATGGCTCTCCAAGTTCAG | TGGCTCACGCTAAGGAAC |
| *OsPORB* | CCGCAAGGAGGGAGCGGTG | CCCTCTTGGTGCTAAGGCCG |
| *OsHd1* | TCAGCAACAGCATATCTTTCTCATCA | TCTGGAATTTGGCATATCTATCACC |
| *OsGI* | GTGGATGCGCTTTGTGACAT | GGCCTGCAGAACGATAGCA |
| *OsEhd1* | GGATGCAAGGAAATCATGGA | AATCCCATCGGAAATCTTGG |
| *OsHd3a* | CTTCAACACCAAGGACTTCGC | TAGTGAGCATGCAGCAGATCG |
| *OsRFT1* | TGACCTAGATTCAAAGTCTAATCCTT | TGCCGGCCATGTCAAATTAATAAC |
| *OsACT1* | TGGCATCTCTCAGCACATTCC | TGCACAATGGATGGGTCAGA |

Tab.S4 Segregation analysis of the *LHD3* allele in several F_2_ populations

| combinations | F_1_ | | F_2_ | | χ^2^(3:1) | P-value |
| --- | --- | --- | --- | --- | --- | --- |
|  | Normal plant NO. | Late heading plant NO. | Normal plant NO. | Normal plant NO. |  |  |
| *lhd3*/NPB | 4 | 0 | 87 | 27 | 0.10 | 0.74 |
| NPB/*lhd3* | 6 | 0 | 142 | 52 | 0.34 | 0.56 |
| *lhd3*/ZH11 | 4 | 0 | 76 | 22 | 0.34 | 0.56 |
| ZH11/*lhd3* | 5 | 0 | 95 | 36 | 0.43 | 0.52 |
| *lhd3*/WYJ7 | 7 | 0 | 162 | 48 | 0.51 | 0.47 |
| WYJ7/*lhd3* | 5 | 0 | 107 | 32 | 0.29 | 0.59 |
